# Remarks on Mr. Martin's Case of Imperforate Anus

**Published:** 1808-05

**Authors:** O. W. Bartley

**Affiliations:** Nailsworth, Glocestershire


### *To the Editors of the Medical and Physical Journal.*

Gentlemen,

I Beg leave to trouble you with a few Observations, in reply to a Query, proposed by Mr. Martin, in his paper on Imperforate Anus, in vol. xviii. p. 298, of your popular Journal. I shall make no remarks on the leading parts of the Case, but that it appears to me to have been judiciously treated, except that I conceive the perforation made by the trochar is not of sufficient extent to afford a proper passage for the fæces, nor is a common sized urethra bougie of sufficient bulk to prevent too close an adhesion. My practice in this disease has not enabled me to afford a decisive answer to his question, "How long the disposition to contract is likely to continue?" Nor, probably, probably, to furnish him with any new or useful information: I shall therefore, if consistent with your plan, give some extracts from writers on that subject, which I hope will prove acceptable to him.

Mr. Petit observes, "that it is very difficult to hit upon the right part for making a perforation into the rectum of children, born without any anus; for he finds, generally, the extremity of the gut formed into a knot. For performing such an operation, he recommends a trochar, the canula and circular plate of which are so slit open as to serve as a groove for a lancet or bistoury to be run in, to enlarge the aperture after the trochar has been pushed into the gut." Mem. de l'Acad. de Chirurg. tom. i.

We have also two Cases of this kind, recorded in Noveau Recueil d'Observations Chirurgicales, par M. Saviard, which I shall fully transcribe.

"Du fondement Clos.

"Le 16 Novembre, 1693, on amena à l'Hotel Dieu, un enfant de quatre jours que avoit une clôture au fondement. J'examinai l'endroit oú l'ouverture naturelle devoit etre, & j'appercus une membrane tendue sur le froncis de l'extremitie de boyau droit, â travers de laquelle. Je distinguai la couleur noire du premier excrement que les enfans rendent par le siege apres leur naissance, que l'on appelle le mæconium. Je n'hesitai point à ouvrir cette membrane avec une bistouri droit, & cette ouverture donna passage à cet excrement noiratre, apres quoi je pansai la playe pendant trois jours, avec une tente enduite d'onguent digestif; afin d'empechir la reunion, & l'enfant se trouve gueri."

"Quelques temps apres on m'en amena un autre auquel l'operation etoit plus incertaine & plus perileuse n'ayant aucune apparence d'anus. Cependant comme cet enfant ne pouvoit pas vivre sans rendre ses excremens. Je disposai aussitot a faire l'operation. Pour cela j'armai une longue lancette dont je me sers pour ouvrir les grands abcés, avec une bandelette afin d'affermir sa lame sur sa chasse; puis je l'enfoncai dans l'endroit ou je jugeai que l'overture auroit du se trouver, & ou je crus pouvoir rencontrer l'intestin l'ayant poussee jusqu'a trois travers de doigts de profondeur, je trouvai le meconium qui coula au long ma lancette, & je senti que son extremité ne trouvoit plus de resistance. Je retirai alors mon instrument, apres avoir elargi l'ouverture de cote & d'autre autant que je le crus necessaire, & je pansai ensuite cette playe comme la precedente, si ce n'est que je fis la tente plus longue; pardessus; pardessus; j'appliquai une compresse trempee dans le vin aromatique, & le bandage en T."

I should have contented myself with simply giving Mr. M. references to the works I have quoted; but considering it possible they might not be in his possession, I trust he will excuse the liberty I have taken in giving extracts from them.

I shall conclude with observing, that I would recommend Mr. M. to enlarge the artificial anus he has made, and to introduce something of larger bulk to prevent a reunion of the parts, until the divided edges have become fully cicatrized.

I am, &c.

O. W. BARTLEY.

*Nailsworth, Glocestershire, April* 4, 1808.

P. S I must apologize for not having made this communication earlier, as probably, ere now, a successful termination of the Case may have rendered the foregoing remarks unnecessary.

